# Generating a transgenic mouse line stably expressing human MHC surface antigen from a HAC carrying multiple genomic BACs

**DOI:** 10.1007/s00412-014-0488-3

**Published:** 2014-10-12

**Authors:** Yoshinori Hasegawa, Tomoyuki Ishikura, Takanori Hasegawa, Takashi Watanabe, Junpei Suzuki, Manabu Nakayama, Yoshiaki Okamura, Tuneko Okazaki, Haruhiko Koseki, Osamu Ohara, Masashi Ikeno, Hiroshi Masumoto

**Affiliations:** 1 Laboratory of Cell Engineering, Department of Frontier Research, Kazusa DNA Research Institute, 2-6-7 Kazusa-Kamatari, Kisarazu, Chiba 292-0818 Japan; 2 Department of Technology Development, Kazusa DNA Research Institute, 2-6-7 Kazusa-Kamatari, Kisarazu, Chiba 292-0818 Japan; 3 RIKEN Center for Integrative Medical Sciences (IMS-RCAI), 1-7-22 Suehiro-cho Tsurumi-ku, Yokohama, Kanagawa 230-0045 Japan; 4 Chromo Research Inc., 1212 Shihongi, Midori-ku, Nagoya, Aichi 458-0039 Japan

## Abstract

The human artificial chromosome (HAC) vector is a promising tool to improve the problematic suppression and position effects of transgene expression frequently seen in transgenic cells and animals produced by conventional plasmid or viral vectors. We generated transgenic mice maintaining a single HAC vector carrying two genomic bacterial artificial chromosomes (BACs) from human HLA-DR loci (DRA and DRB1). Both transgenes on the HAC in transgenic mice exhibited tissue-specific expression in kidney, liver, lung, spleen, lymph node, bone marrow, and thymus cells in RT-PCR analysis. Stable functional expression of a cell surface HLA-DR marker from both transgenes, DRA and DRB1 on the HAC, was detected by flow cytometric analysis of splenocytes and maintained through at least eight filial generations. These results indicate that the de novo HAC system can allow us to manipulate multiple BAC transgenes with coordinated expression as a surface antigen through the generation of transgenic animals.

**Electronic supplementary material:**

The online version of this article (doi:10.1007/s00412-014-0488-3) contains supplementary material, which is available to authorized users.

## Introduction

Transgenic animals have provided tools for investigating many biological problems. Genomic fragments cloned by bacterial artificial chromosomes (BACs) have been utilized to generate transgenic animals when tissue-specific or temporally controlled expression of transgenes is desired. Due to the large insert capacity (∼350 kb) of a BAC vector, the genomic fragments can often possess the complete promoters and control elements of the gene of interest (Asami et al. 2011). In addition, BAC transgenes seem to be more resistant to position effects than smaller transgenes, such as artificial expression cassettes with complementary DNA (cDNA) (Gong et al. 2003). Typically, BAC transgenic mice are generated by microinjection of the BAC DNA into the pronucleus of fertilized mouse eggs (Vintersten et al. 2008). However, in principle, this method causes random integration (non-specific insertion) of BAC DNAs into the mouse genome, and the number of insertion copies is variable. Increased copy number of a BAC transgene correlates with increased expression of the BAC transgene (Chandler et al. 2007). When investigating the cooperation of two transgenes in a transgenic mouse, generally two characterized transgenic mouse lines are crossed, but this is a time-consuming method and maintaining an appropriate level of gene expression is difficult.

A de novo human artificial chromosome (HAC) was constructed with naked human centromeric repetitive DNA (Harrington et al. 1997; Ikeno et al. 1998) and a HAC vector system developed in which one copy of a DNA fragment can be handled by Cre/lox insertion and transferred into a variety of vertebrate cell lines (Ikeno et al. 2009; Iida et al. 2010). A HAC is an episomal vector that can harbor a large DNA and is exploitable for generating transgenic animals using embryonic stem (ES) cell technology (Kazuki and Oshimura 2011; Ikeno et al. 2012). Thus, the HAC system can avoid the copy number problem and/or position effects caused by non-specific insertion of the BAC transgene. The HAC vector is expected to be available for the production of transgenic mice carrying two or more single-copy genes with a large control region over tens of kilobases. Recently, a transgenic mouse harboring a single copy of a HAC, termed a trans-mini-chromosomal (TMC) mouse, carrying three continuous non-correlated genes from the human genome was generated by inserting a single BAC DNA from chromosome 21 (Miyamoto et al. 2014). However, whether two or multiple independent BAC transgenes can be gathered onto a single HAC and cooperatively function in a transgenic mouse has not been investigated.

Here, we describe a transgenic mouse using a HAC vector carrying two single-copy human HLA-DR genomic genes. HLA-DR is a major histocompatibility complex (MHC) class II cell surface receptor consisting of an αβ heterodimer. We introduced a DR α-chain (HLA-DRA locus) and DR β-chain (HLA-DRB1*0405 locus) into a single HAC vector (HLA-HAC). In transgenic mice harboring HLA-HAC (carrying HLA-DRA and DRB1 genes), tissue-specific expression of human MHC class II cell surface receptor in spleen cells was detected by flow cytometric analysis through at least eight filial generations.

## Materials and methods

### Cell culture

Chinese hamster ovary (CHO) cells were cultured in Ham’s F-12 nutrient mixture (Wako) supplemented with 10 % fetal bovine serum (FBS) at 37 °C and 5 % CO_2_. The mouse ES cells were maintained on feeder cells in an ES cell medium consisting of Dulbeccos’ modified Eagle’s medium (DMEM) (Kohjin Bio) supplemented with 20 % FBS, 0.1 mM non-essential amino acids (Gibco), 2 mM glutamine (Gibco), 1000 U/ml ESGRO (Chemicon), and 0.1 mM β-mercaptoethanol (Sigma).

### Microcell-mediated chromosome transfer

Microcell-mediated chromosome transfer (MMCT) from CHO cells to mouse ES cells was carried out as described previously (Suzuki et al. 2006). Briefly, twenty 10-cm dishes of CHO cells were grown to 70 % confluency and Colcemid (Wako) added to 0.05 μg/ml. The cells cultured for 72 h were harvested by trypsinization and resuspended in pre-warmed serum-free DMEM (Wako) containing Cytochalasin B (Calbiochem) at a final concentration of 20 μg/ml. The suspension was incubated, and then an equal volume of Percoll (Amersham Biosciences) was added. The suspension was centrifuged in a Hitachi R20A2 rotor at 15,000 rpm for 90 min at 37 °C. The microcell fraction containing the HLA-HAC was mixed with ES cells. After centrifugation at 2000 rpm for 5 min, the pellet was suspended in 1 ml 50 % PEG1500 (Roche). The fusion product was washed and plated onto three 10-cm dishes layered with feeder cells. ES cells containing the HLA-HAC were selected with 150 μg/ml G418 (Sigma).

### Generation of chimeric mice

Chimeric mice were produced from mouse ES cell lines. The ES cells were aggregated with eight-cell embryos derived from BDF2 mice and then transferred into pseudopregnant ICR females. Almost 100 % coat color chimeric mice were mated with C57BL/6 (B6) mice (Jackson Laboratory) to obtain transgenic mice. All animal experiments were approved by the Institutional Animal Care and Use Committee of RIKEN RCAI and Kazusa DNA Research Institute.

### HLA-DR gene constructs

**Fig. 1:**
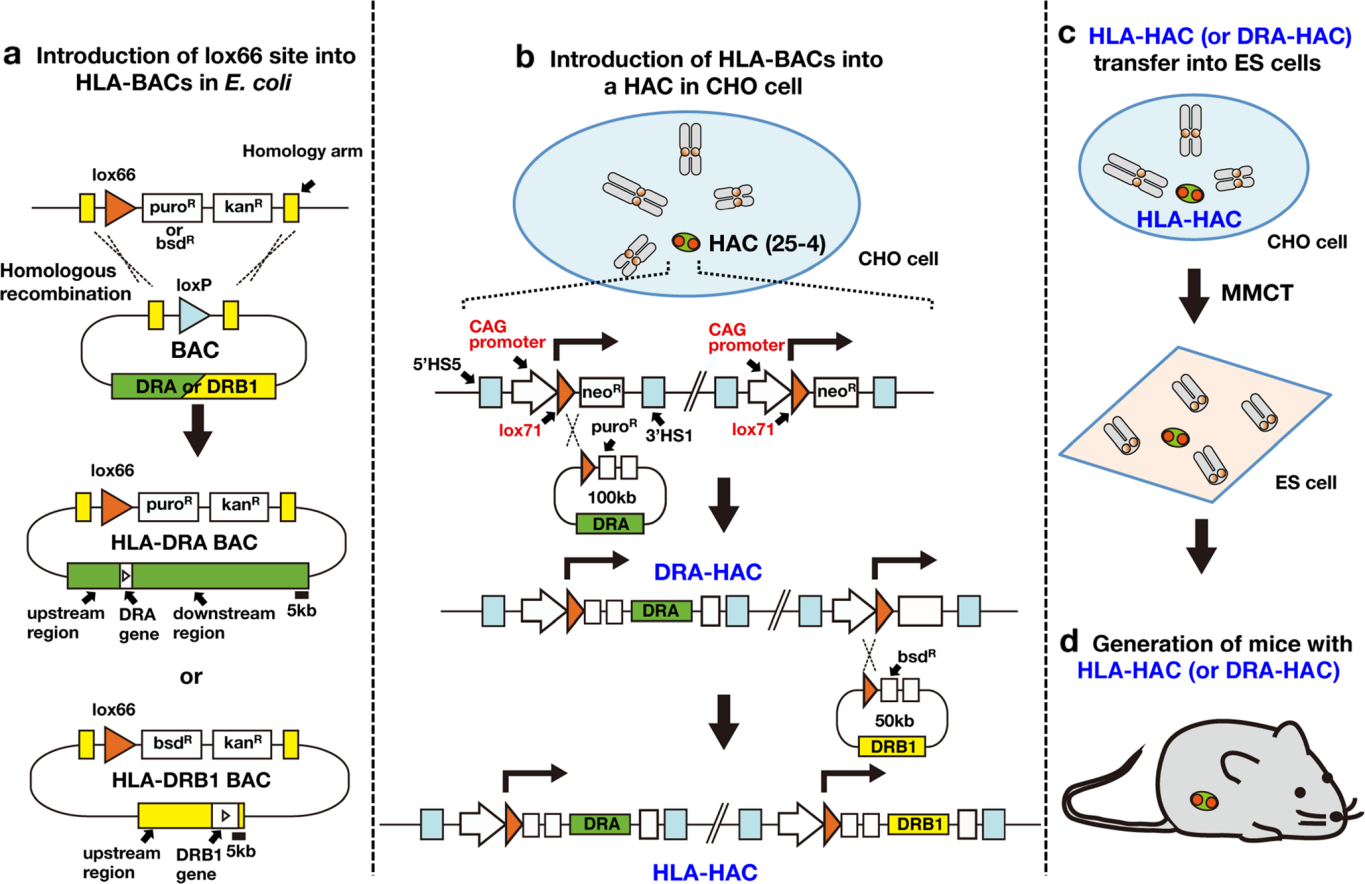
Generation of mice with HACs containing human genes. **a** Introduction of the lox66 site into the HLA-BAC by Red recombination in *E. coli*. **b** Schematic representation of two consecutive introductions of HLA-BACs into a HAC vector using Cre/lox recombination. The gene in the entry vector was inserted into a HAC vector in CHO cells by Cre/lox recombination. Successful recombinants were selected by puromycin (*puro*) or blasticidin (*bsd*) resistance. **c** HLA-HAC transfer from CHO cells to mouse ES cells was achieved by MMCT. **d** Chimeric mice with HLA-HAC were created by aggregation of the ES cells with BDF2 eight-cell embryos

We used a whole CTD-2052L14 BAC clone (100 kb) as the HLA-DRA gene because this BAC contains only the HLA-DRA gene (referenced on NCBI CloneDB browser; http://www.ncbi.nlm.nih.gov/clone/79827/). The DRA-BAC used in this study contains an upstream 22-kb region and the HLA-DRA gene (Fig. 1a). Only a 268-base pair region of the 5′-flanking region of the HLA-DRA gene has been reported to be sufficient for the cell type-specific expression on the transgenic mouse as a multi-copy in this case (Fukui et al. 1993). So, we included the longer upstream region. On the other hand, for the *cis*-elements of the DRB1 gene, consensus has not yet been reached; therefore, we used the maximum length of the upstream region, close to the border of the next pseudo gene. A HLA-DRB1*0405 BAC was constructed from a 28.5-kb upstream region of the HLA-DRB1*0103 gene in a RP11-379F19 BAC clone (referenced on NCBI CloneDB browser; http://www.ncbi.nlm.nih.gov/clone/312446/) and genomic DNA (IHW09415) containing all exons of HLA-DRB1*0405 by using a Red recombination with pBADTcTypeG plasmids whose mutant Redα and mutant Redβ proteins increase the recombination efficiency (Nakayama and Ohara 2005). Consequently, the 50-kb BAC had 28.5 kb upstream of the translational start site and all exons and introns of the HLA-DRB1*0405 gene (Fig. 1a). The HLA-DRA and DRB1 BACs were modified by addition of the lox66/puromycin resistance cassette or lox66/blasticidin S resistance cassette, respectively (Fig. 1), in place of loxP by the Red recombination in *Escherichia coli* according to a previously described method (Ikeno et al. 2009).

### DNA transfection

For the insertion of the HLA-DR BACs into the HAC vector, 1 μg of the HLA-DR BAC DNA was co-transfected with 0.5 μg of CAGGS-Cre into CHO cells (5 × 10^5^) retaining the HAC vector (Ikeno et al. 2009) with FuGENE HD (Promega) according to the manufacturer’s instructions. DRA cell lines were selected with 6 μg/ml puromycin (Sigma) and DRB1 cell lines with 3 μg/ml blasticidin S (Wako).

### Real-time quantitative PCR analysis

Total RNA was isolated using the RNeasy Micro Kit (Qiagen). cDNA was synthesized using the Verso cDNA Synthesis Kit (Thermo Scientific), and 25-ng aliquots were used for PCR. Real-time PCR was carried out using the ABI 7500 Real-Time PCR System (Applied Biosystems). PCR reactions were carried out using the Luminaris Probe Low ROX qPCR Master Mix (Thermo Scientific) and each assay mix: Hs00219578_m1 (HLA-DRA), Hs00830030_sH (HLA-DRB1), or Mm99999915_g1 (mouse GAPDH). The PCR protocol was 95 °C for 10 min and 40 cycles of 95 °C for 15 s and 60 °C for 60 s.

### Genomic PCR analysis

Genomic DNA was extracted from mouse tails using a Wizard Genomic DNA Purification Kit (Promega) and PCR performed as follows. The amplification conditions were 98 °C for 1 min, followed by 35 cycles of 98 °C for 10 s, 60 °C for 30 s, and 72 °C for 30 s. The following primers were used: HLA-DRA-1, 5′-CACGAACAGCCCTGTGGAAC-3′ and 5′-CTCAGTTGAGGGCAGGAAGG-3′; HLA-DRA-2, 5′-TCTCCCAGAGACTACAGAGAACG-3′ and 5′-CCTGCGTTCTGCTGCATTG-3′; HLA-DRB1-1, 5′-TCATTTCTTCAACGGGACGGAG-3′ and 5′-TGCACTGTGAAGCTCTCACC-3′; HLA-DRB1-2, 5′-TGGTCTGCTCTGTGAATGG-3′ and 5′-TCCACTGTGAGAGGGCTCATC-3′; HLA-DRB1-3, 5′-GTGGGAGATGCAGACTTGTGG-3′ and 5′-TGCTTCTTCCTCATCATCTCTGC-3′; and HLA-DRB1-4, 5′-GAACCAACACACCGACGGATAG-3′ and 5′-GAGGGATTGGACACAGAGATCAG-3′.

### Fluorescent in situ hybridization

Fluorescent in situ hybridization (FISH) analysis was carried out according to conventional procedures. To detect HACs, biotin-labeled α21-I alphoid DNA (11-4) (Ikeno et al. 1994) and digoxigenin-labeled pBelo-BAC were used as probes. For dual FISH, biotin-labeled DNA was visualized with FITC-conjugated avidin (Vector) and digoxigenin-labeled DNA with TRITC-conjugated antidigoxigenin antibody (Roche).

### Preparation of spleen cells

Splenocytes were smashed with two slide glasses, filtered through a 70-μm cell strainer, and washed with Roswell Park Memorial Institute (RPMI) medium (Wako). The supernatant was removed and the red blood cells were destroyed with Red Blood Cell Lysing Buffer Hybri-Max (Sigma). Cells were washed with RPMI medium containing 10 % FBS and then resuspended in the same medium.

### Flow cytometry

Cells were stained with the antibody against HLA-DR conjugated with Alexa Fluor 488 (50 μg/ml, L243, BioLegend) for 30 min on ice. Flow cytometric analysis was performed using a FACS Calibur instrument (BD Biosciences) and the results analyzed using the FlowJo software program (Tree Star).

## Results

### Introduction of HLA-BACs into a HAC vector in CHO cells

**Fig. 2:**
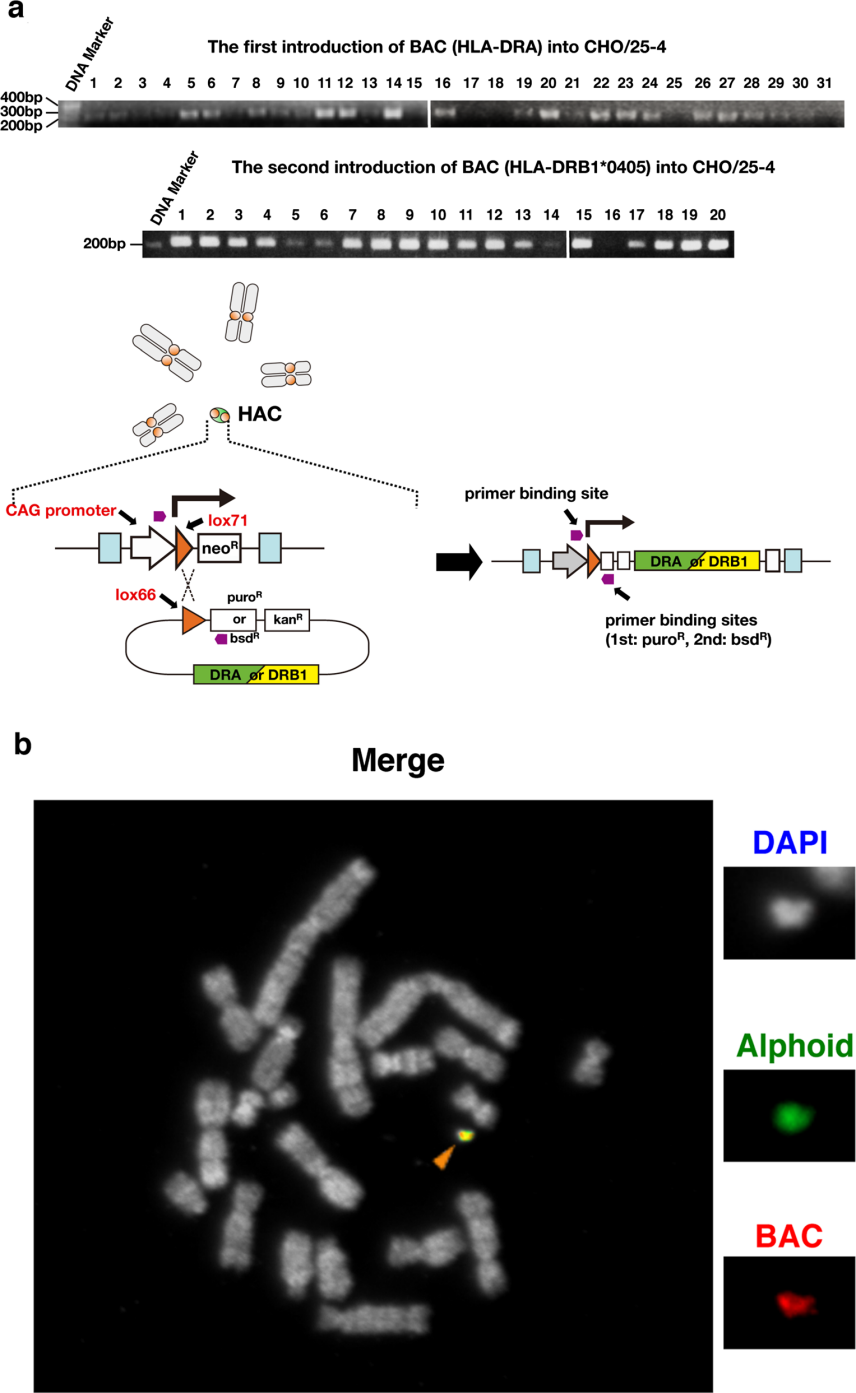
HLA-BAC introduction into a HAC vector in CHO cells. **a** PCR analysis of two consecutive introductions of HLA-BACs into a HAC vector using site-specific recombination. The first and second introductions of BAC were confirmed by PCR with the CAG promoter and puro sequences (22 positive in 31 analyzed) and the CAG promoter and bsd sequences (19 positive in 20 analyzed), respectively. **b** FISH analysis of the HLA-HAC (*arrowhead*) in CHO cells. This HAC carried a single copy of HLA-DRA BAC and a single copy of HLA-DRB1 BAC, confirmed by a further insertion of a DNA fragment to the remaining cassettes on the HAC and PCR analysis (data not shown). The *red signal* shows BAC DNA, the *green signal* shows alphoid DNA and the *white signal* shows DNA counterstained with DAPI

The HLA-BACs with lox66/puromycin or blasticidin S resistance cassette were inserted into a HAC vector in CHO cells by Cre/lox recombination. Successful Cre-lox recombination between the lox66 site at the promoterless cassette (puro or bsd) on the BAC and the lox71 site of the gene expression cassette on the 25-4 HAC vector produced drug-resistant cell lines (puro or bsd; Fig. 1). PCR analysis of drug-resistant cells after the first insertion of the HLA-DRA BAC into the 25-4 HAC showed that 22 of 31 cell lines had successful insertion. The second insertion of the HLA-DRB1*0405 BAC was performed using the first insertion-positive cell line (No. 11; Fig. 2a). The 25-4 HAC vector carrying HLA-DRA BAC (DRA-HAC) and carrying both HLA-DRA BAC and HLA-DRB1*0405 BAC (HLA-HAC) was stably maintained as an extra chromosome through Cre-lox reactions in CHO cells (Fig. 2b).

### Transfer of the HACs into mouse ES cells by MMCT

**Fig. 3:**
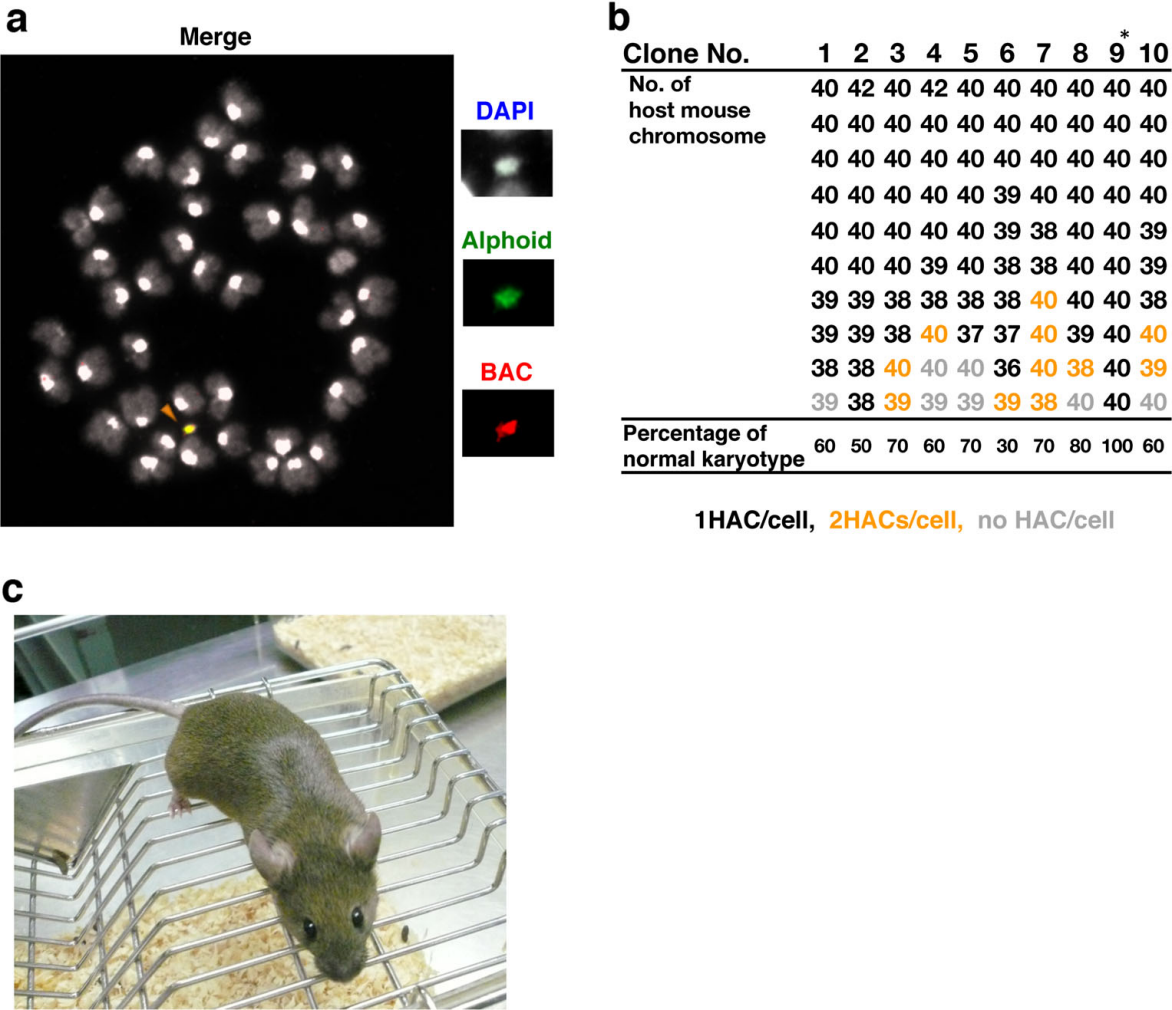
Generation of TMC mice. **a** FISH analysis of the HLA-HAC in mouse ES cells. The mouse ES cell lines carrying HLA-HAC (*arrowhead*) were established by MMCT. The *red signal* shows BAC DNA, the green signal shows alphoid DNA and the *white signal* shows DNA counterstained with DAPI. **b** Karyotype analysis of mouse ES cells harboring HLA-HAC. An *asterisk* (*) indicates the clone used for generation of the TMC mouse. **c** Chimeric mouse from mouse ES/HLA-HAC. The coat color of the mouse ES cell is agouti. This mouse is almost 100 % ES cell-derived

We used mouse ES cells (1 × 10^7^ cells) and microcells prepared from CHO cells (1 × 10^7^ cells) containing DRA-HAC or HLA-HAC for the MMCT (Doherty and Fisher 2003, Fig. 1c). We obtained over 70 mouse ES cell colonies screened by G418 resistance and analyzed 10 colonies by FISH. The majority of each ES cell lines carried a single copy of the HAC with no integration signal of the HAC DNA in the mouse chromosomes (Fig. 3a, b). Karyotype analysis showed that the number of cells with a normal mouse karyotype (retaining 40 mouse chromosomes) varied between 30 and 100 % among ES cell lines (Fig. 3b). We used cell line No. 9 because 10 of 10 analyzed cells showed normal karyotypes and contained a single HLA-HAC for chimeric mouse production.

### Chimeric mice carrying the HACs

Chimeric mice carrying the DRA-HAC or HLA-HAC were created by aggregation of the ES cells with BDF2 eight-cell embryos. Three male mice with a high degree of chimerism (almost 100 % in coat color, Fig. 3c) were investigated for germ line transmission of the HLA-HAC. After crossing the chimeric male mice with five female B6 mice, 6-week-old F1 offspring (39 F1 mice) were analyzed by PCR using HAC-specific primers; 35.3–58.3 % of these F1 offspring were positive for the HAC signals (Table 1). Thus, HLA-HACs were transmitted through the germ line of all of the chimeric mice we examined, and a total of 46.2 % of F1 offspring (18 of 39) possessed the HLA-HAC (maximum expected value was 50 %). On the other hand, three chimeric male mice harboring the DRA-HAC were crossed with six female B6 mice; 47.1–53.3 % of these F1 offspring were positive for the HAC signals (Table 1). A total of 49.1 % of F1 offspring (26 of 53) possessed the DRA-HAC. Because the ES cell line used in this experiment was selected carefully by karyotype analysis and HAC retention rate, we effectively obtained chimeric mice with chimeric rates and HAC transmission rates that were high enough.

**Table 1:** HAC transmission rate for six chimeric mice

Parental origin	Total offspring	Total HAC+ mice	HAC+ mice/offspring (%)
HLA-HAC male No. 1	12	7	58.3
HLA-HAC male No. 2	10	5	50.0
HLA-HAC male No. 3	17	6	35.3
HLA-HAC male total	39	18	46.2
DRA-HAC male No. 4	15	8	53.3
DRA-HAC male No. 5	17	8	47.1
DRA-HAC male No. 6	21	10	47.6
DRA-HAC male total	53	26	49.1

### Mitotic stability of the HACs in mouse somatic tissues

**Fig. 4:**
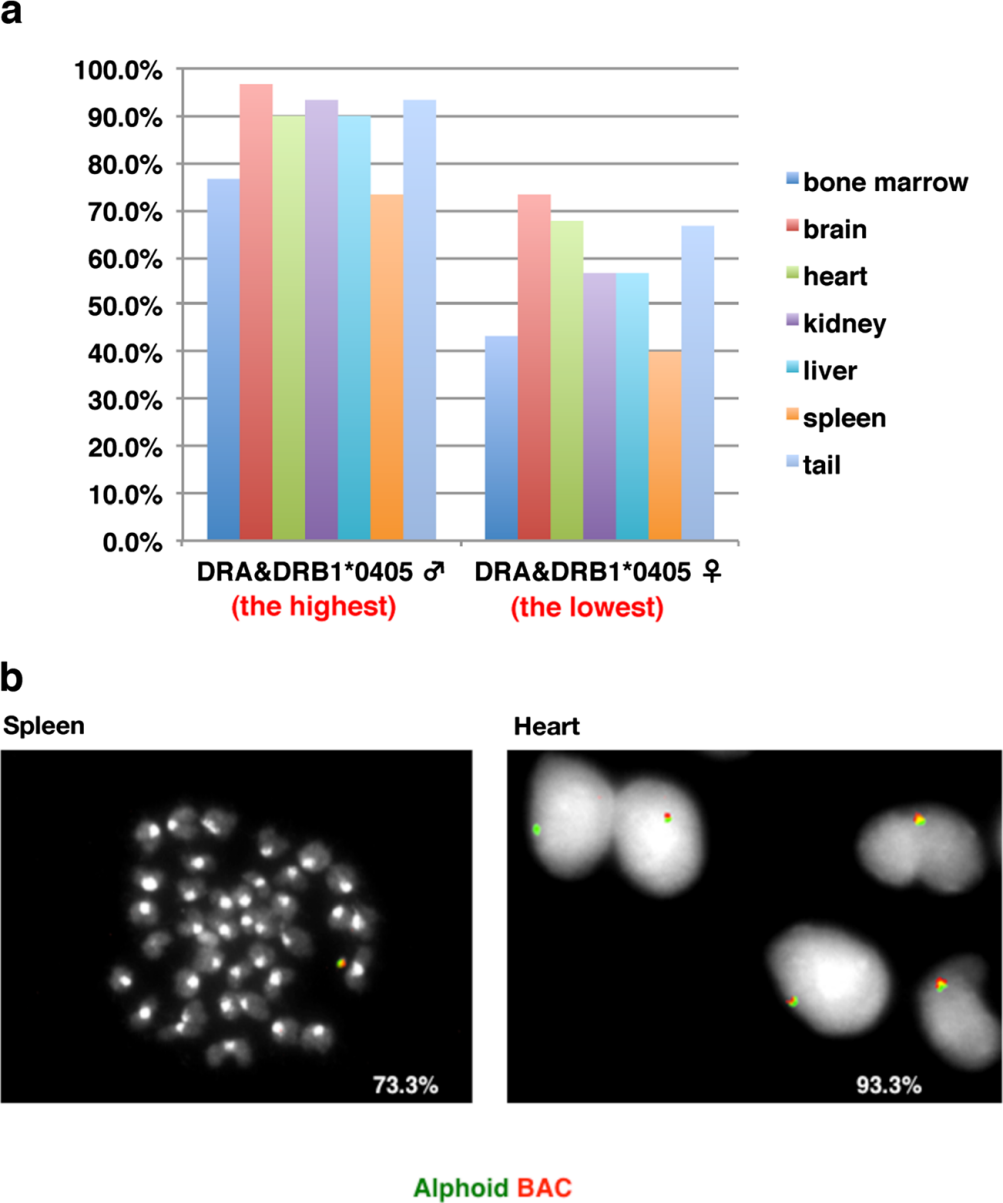
Characteristics of transgenic mice harboring HLA-HAC. **a** HLA-HAC retention rate in the bone marrow, brain, heart, kidney, liver, spleen, and tail of a 7-month-old F1 mouse. The percentage of HAC-retaining cells was calculated from 60 cells. **b** Metaphase chromosomes from the spleen and interphase nuclei from the heart of a 7-month-old F1 mouse (the highest HAC retention male in Table 2) were analyzed by probing for alphoid and BAC DNA in FISH

To investigate the mitotic stability of HACs in the somatic tissues of mice, the retention of HLA-HAC in cells from the bone marrow, brain, heart, kidney, liver, spleen, and tail of 7 months old F1 mice was analyzed by FISH. As expected, the HLA-HACs were retained in all of these tissues (Fig. 4a, b). The retention rate in the brain, heart, kidney, liver, and tail at 7 months (highest individual (male) 90.0–96.7 %, lowest individual (female) 56.7–73.3 %) was similar to that of tail cells from a 6-week-old mouse (highest individual 96.7 %, lowest individual 60.0 %; Table 2). In contrast, the HAC retention rate of the bone marrow and spleen was lower than that of the other tissues (highest individual 73.3 and 76.7 %, lowest individual 43.3 and 40.0 %, respectively). The HAC retention rates in the tissues varied among F1 mice, but they were represented in tail cells.

**Table 2:** HAC retention rate in the tail cells of 6 weeks old F1 HLA-HAC mice

	HAC retention rate (%)	Sex of offspring	Parental origin
	60.0	♀^b^	No. 2♂
	66.7	♂	No. 1♂
	66.7	♀	No. 2♂
	73.3	♂	No. 3♂
	76.7	♀	No. 3♂
	76.7	♂	No. 1♂
	80.0	♂^a^	No. 2♂
	83.3	♂	No. 3♂
	86.7	♀	No. 3♂
	86.7	♀	No. 3♂
	86.7	♂	No. 3♂
	86.7	♂	No. 1♂
	90.0	♀	No. 1♂
	90.0	♀	No. 2♂
	90.0	♂	No. 2♂
	93.3	♀	No. 1♂
	96.7	♂^b^	No. 1♂
	96.7	♂	No. 1♂
Average	82.6		

^a^The first mouse used for HAC transmission through eight generations

^b^Mice used for analyzing the HAC retention rate in the bone marrow, brain, heart, kidney, liver, spleen, and tail of 7-month-old F1 mice (see also Fig. 4a, b)

### Retention and transmission of the HACs through mouse generations

**Fig. 5:**
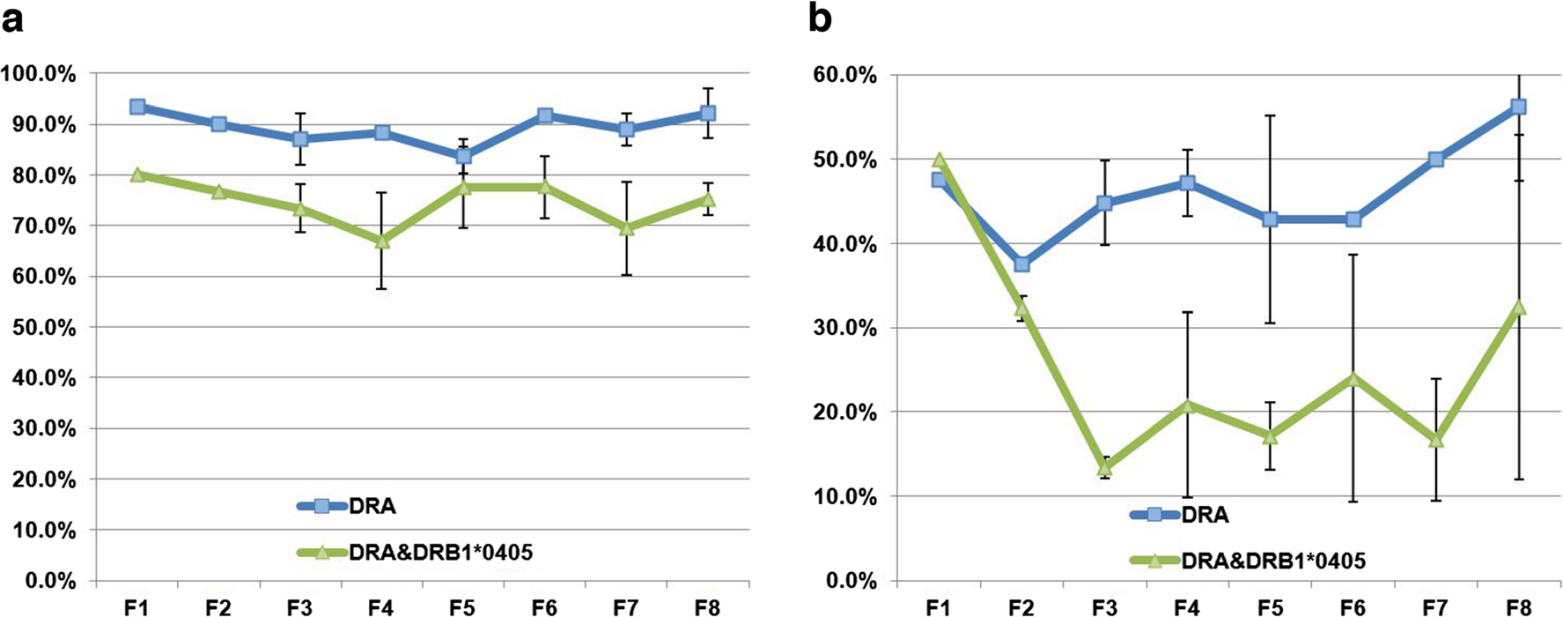
The retention and transmission rates of HAC. **a** The retention rate of HAC in tail cells was determined by FISH analysis using alphoid and BAC DNA probes. The percentage of HAC-retaining cells was calculated from 60 cells. *Error bars* indicate standard deviations. **b** An average of each transmission rate was determined by more than two crossings

To investigate the retention and transmission rates of HACs in the TMC mouse and the stability of the large insert genes on the HAC through mouse generations, F1 mice were backcrossed with B6 mice for seven more generations for a total of eight backcrosses. “The HAC transmission” of all pups was examined by PCR analysis of genomic DNA extracted from the 3-mm tail of each mouse with HAC-specific primers. “The retention rate of the HAC” in tail cells from the PCR positive mouse was examined by FISH analysis. (The PCR band-negative mice did not show any HAC FISH signal.) In F1 mice, the retention rates of HLA-HAC and DRA-HAC were similar on average but variable in individual mice (average 82.6 and 86.2 %, respectively; Tables 2 and 3). The retention rate of HLA-HAC in tail cells through mouse generations was 70–80 %, whereas the retention rate of DRA-HAC was around 90 % (Fig. 5a). Unexpectedly, a significant difference was observed in the transmission rate between the two types of HACs through mouse generations. The transmission rate of DRA-HAC was very close to the maximum expected value of 50 % (average 45.9 %, 61/133 mice) through eight generations, but the transmission rate of HLA-HAC varied in lower ranges (average 23.5 %, 38/162 mice, Fig. 5b). Clearly, the additional insertion of the DRB1 BAC into another gene expression cassette site on the HLA-HAC caused some instability in the HAC through the mouse generations. However, all HACs were detected as extra-chromosomal signals independently from endogenous mouse chromosomes, though we observed over 6000 cell samples in the FISH analyses. We also confirmed by genomic PCR that the structures of the HLA genes on the HACs were stably maintained through HAC transfer into the mouse and eight consecutive mouse generations (Supplemental Fig. S1).

**Table 3:** HAC retention rate in tail cells from 6-week-old F1 DRA-HAC mice

	HAC retention rate (%)	Sex of offspring	Parental origin
	63.3	♂	No. 4♂
	66.7	♂	No. 6♂
	76.7	♀	No. 4♂
	80.0	♀	No. 5♂
	80.0	♀	No. 6♂
	83.3	♀	No. 4♂
	83.3	♂	No. 4♂
	83.3	♂	No. 5♂
	83.3	♀	No. 6♂
	83.3	♂	No. 6♂
	86.7	♂	No. 4♂
	86.7	♂	No. 5♂
	90.0	♀	No. 4♂
	90.0	♀	No. 5♂
	90.0	♀	No. 5♂
	90.0	♂	No. 5♂
	90.0	♂	No. 5♂
	90.0	♀	No. 6♂
	90.0	♀	No. 6♂
	90.0	♂	No. 6♂
	93.3	♀	No. 4♂
	93.3	♂	No. 4♂
	93.3	♀	No. 6♂
	93.3	♀	No. 6♂
	93.3	♂^a^	No. 6♂
	96.7	♂	No. 5♂
Average	86.2		

^a^The first mouse used for HAC transmission through eight generations

### Functional expression of HLA-DR genes from the HLA-HAC

**Fig. 6:**
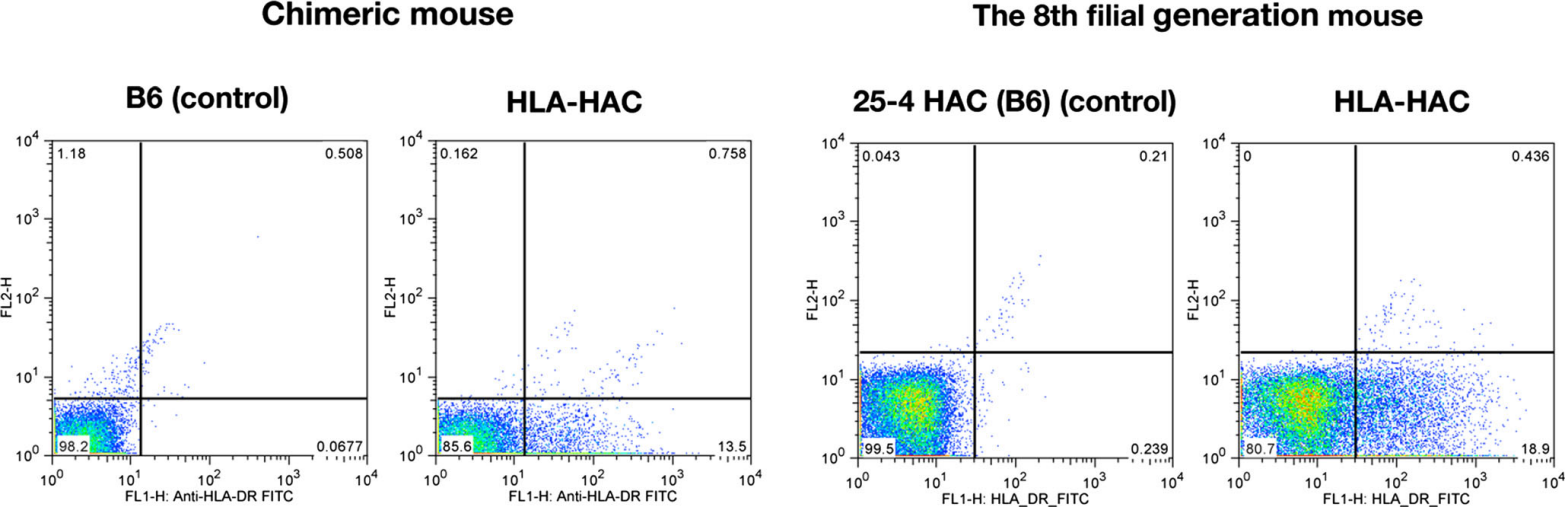
Flow cytometric analysis of surface molecules on splenocytes from a chimeric mouse and F8 generation mouse. Cells were stained with anti-HLA-DR, L243. A B6 mouse with no HAC was used as a negative control for the chimeric mouse, and the B6 background for five filial generations with HAC (no inserts) was used as a negative control for the F8 generation mouse

The expression of HLA-DRA and HLA-DRB1 as cell surface antigens was evaluated by flow cytometry using splenocytes from chimeric mice and anti-HLA-DR antibody. The cell surface signals were detected specifically from the chimeric mouse carrying the HLA-HAC (13.5 % of cell population, Fig. 6a). Because the antibody binds a conformational epitope on HLA-DRα that depends on the correct folding of the αβ heterodimer (Moro et al. 2005), the result clearly indicates that the HLA-DRA and HLA-DRB1*0405 genes from the HLA-HAC were expressed and folded as a αβ heterodimer on the cell surface of splenocytes in the chimeric mouse. Functional expression was also observed in the eighth-generation TMC mice (18.9 % of cell population, Fig. 6b), indicating that such functional expression of the human genes on the HAC was maintained through mouse generations. It was reported that only about a half population (51.6 %) of splenocytes was expressing endogenous class II MHC on their cell surface by flow cytometric analysis in B6 wild-type mice (Jux et al. 2013). According to this data, an expected population of the splenocytes expressing the HLA from the HAC in the chimeric mouse is estimated to be approximately 20–37 % (40–73 % splenocytes retain the HLA-HAC; Fig. 4a) and that in F8 mouse is approximately 22–30 % (44–60 % splenocytes retain the HLA-HAC estimating from the 74 % HAC retention rate in the tail cells; Fig. 5a). The actual measurements of the cell surface signals by flow cytometry using splenocytes are 13.5 % in the chimeric mouse and 18.9 % in the F8 mouse. These values, more than a half or very close to the expected values estimated from mouse endogenous class II MHC expression, are significant enough for considering transgenic human class II MHC genes on the HAC vector in mice through generations.

### Tissue-specific expression of HLA-DR genes from the HAC in mice

**Fig. 7:**
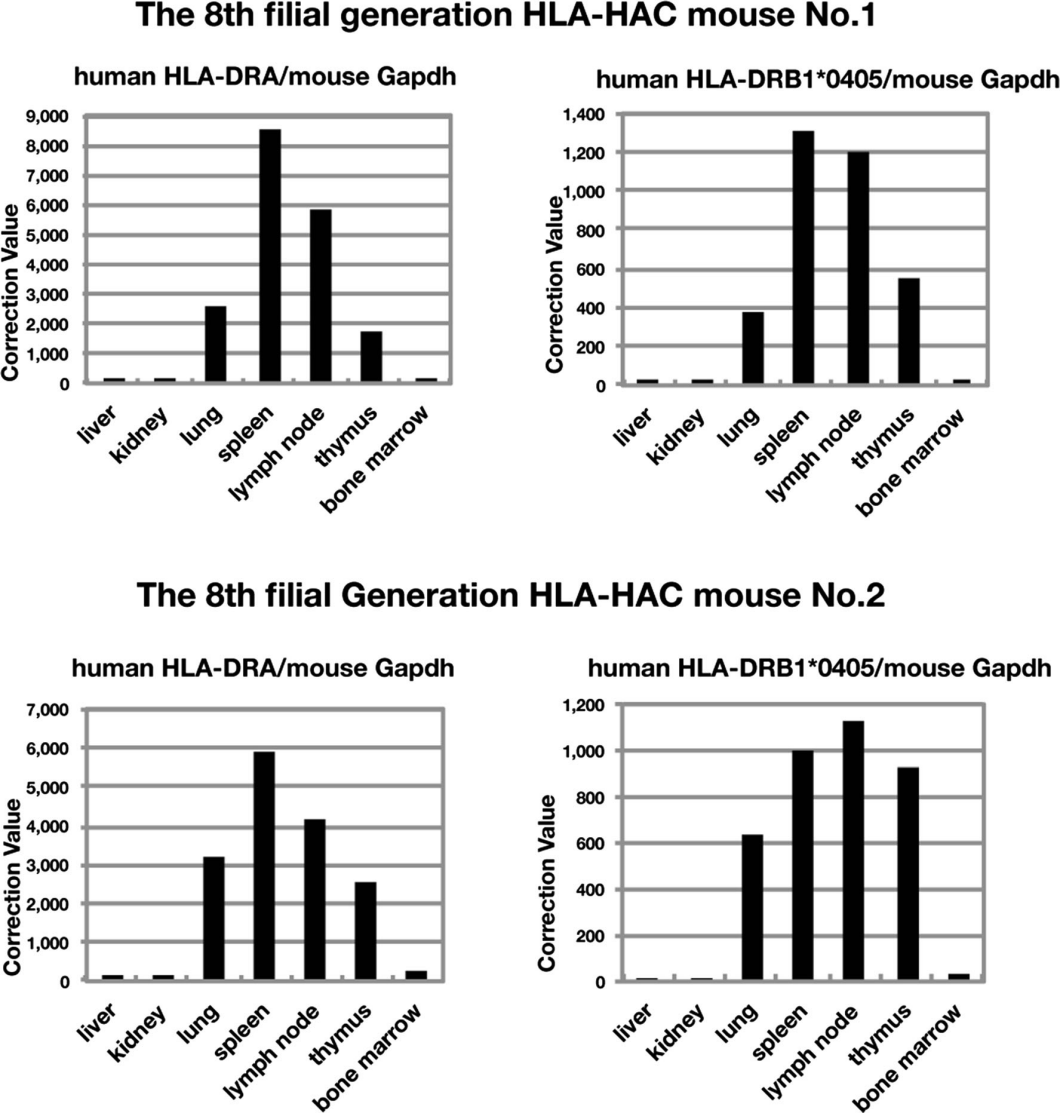
Quantitative analysis of the expression of human transgenes in various tissues of TMC F8 mice. Each value was corrected by the level of mouse GAPDH (see Supplemental Fig. S2)

The expression of MHC class II molecules is basically confined to immunocompetent cells, such as B cells, macrophages, dendritic cells, and thymic epithelial cells. To determine whether the HLA-DR genes on the HAC were expressed in a tissue-specific manner in eighth-generation TMC mice, total cellular RNA was extracted from various tissues and examined by real-time quantitative PCR analysis. The results show that the expression pattern was similar between the two individuals (Fig. 7 and Supplemental Fig. S2). The expression levels of the HLA-DRA and HLA-DRB1 genes were high in the lung, spleen, lymph node, and thymus, but they were extremely low in the liver, kidney, and bone marrow. These results indicate that the natural patterns of tissue-specific expression of the human HLA-DRA and HLA-DRB1*0405 genes were almost mimicked on the HAC through the mouse generations. On the other hand, the expression pattern of these two genes on the HAC in CHO and ES cells was stochastic (Supplemental Fig. S3). The No. 9 ES cell line used for chimeric mouse production showed little, if any, HLA-DRA and HLA-DRB1*0405 expression. Thus, human HLA-DR genes including regulatory elements and promoters used in this study acquired precise expression epigenetically in specific cell types through the mouse developmental process.

## Discussion

We generated transgenic mice using a HAC carrying two single-copy HLA-DR genes. For the past 20 years, many HLA-DR transgenic mice, including those with an immunodeficient NOG background, have allowed the analysis of HLA-restricted responses with variable success (Fukui et al. 1993; Suzuki et al. 2012). This study is the first report of transgenic mice carrying two single-copy human genes composed of human regulatory elements and exon-intron structures. Both the HLA-DRA and HLA-DRB1 genes on the HAC were expressed in a tissue-specific manner and exhibited functional expression as a cell surface receptor in the TMC mouse. The tissue-specific expression pattern of HLA-DR in the TMC mouse was similar to that of endogenous class II MHC genes in mice. The mRNA expression level of the class II MHC in mice was high in the spleen and thymus, but it was barely detectable in the liver, kidney, and bone marrow (Pinkert et al. 1985). A previous report of a mouse carrying 12 copies of 24 kb of the HLA-DRA gene showed that the transgene was overexpressed in some tissues and moderately in other tissues (Altmann et al. 1993). In another transgenic mouse, a 6-kb HLA-DRA gene fragment was integrated into the X chromosome and subjected to random inactivation (Fukui et al. 1993). Based on these observations, our present analysis indicates that the features of HAC, an episomal vector with a large DNA capacity and a controllable gene number, were exploited effectively for the production of HLA-DR transgenic mice. Furthermore, HLA-HACs were transmitted through at least eight generations, and HLA-DR genes on the HAC maintained functional expression during that time. We also confirmed that two human genes that were reorganized on the HAC were cooperatively expressed in immunocompetent cells in mice. Taking account of these results, although we checked only small fragments of the genes on the HAC by PCR (Supplemental Fig. S1), it is conceivable that at least no major rearrangement influencing the functional expression was observed on the HACs through eight mouse generations.

There are over 40 haplotypes of HLA-DRB, but only one functional type of HLA-DRA in the Japanese population. We have already generated a DRA-HAC mouse that harbors a HAC vector carrying only an HLA-DRA locus. Recently, the novel transgenic technique pronuclear injection-based targeted transgenesis (PITT) was reported (Ohtsuka et al. 2010; Ohtsuka et al. 2012). The PITT technique enables targeted integration of a single-copy transgene into a predetermined locus by Cre-loxP-mediated recombination and guarantees a stable and reproducible transgene expression. A combination of the DRA-HAC mouse and such a technique will allow the generation of more HLA-DR haplotypes.

The HAC retention rate of tails was almost the same for the same individual at 6 weeks and 7 months of age (Table 2, Fig. 4a), and the HAC retention rate of the tail at 7 months was the same as that of the brain, heart, kidney, and liver at 7 months. The HAC retaining an early developmental cell proliferation stage in the TMC mouse may determine the consequent HAC retention rate in the tail, brain, heart, kidney, and liver. Thus, the HAC retention rate of the tail cells from young mice is a good and easy index for estimating the retention rate of these tissues. On the other hand, the retention rate of the bone marrow and spleen was relatively lower at 7 months of age. Whether the retention rate of the bone marrow and spleen will decrease with proliferating dependency requires further investigation.

Although the retention rates in the F1 mice harboring the DRA-HAC and the HLA-HAC were variable in individual mice, they were not significantly different on average (86.2 and 82.6 %, respectively). No significant decrease in stability was observed in the previous analysis after inserting three genes at different sites in the expression cassettes on the same HAC vector when evaluating cultured cells (data not shown). However, through the generations, significant differences were observed in the transmission rates between mice carrying the DRA-HAC and HLA-HAC. The DRA-HAC was generated by insertion of a 100-kb HLA-DRA BAC into one of four expression cassettes of the 25-4 HAC vector, whereas the HLA-HAC was generated by insertion of an additional 50-kb HLA-DRB1 BAC into another expression cassette. This additional BAC insertion resulted in a significant decrease in the transmission rate through mouse generations and influenced the meiotic process in the germ line. The expression patterns and levels of HLA-DRA and HLA-DRB1 from the HAC vector were not significantly different and were restricted to immunocompetent mouse cells through the eight generations. Thus, the gene product from the additional HLA-DRB1 gene is not likely to be more harmful in the germ line. The additional BAC insertion at the separated expression cassette site on this circular HAC vector may have some more structural disadvantage through the meiotic process in the germ line. Indeed, we did not observe such stability differences between the DRA-HAC and the HLA-HAC through the HAC constructions in CHO cells and the HAC transfer until generation of chimeric mice. The additional BAC insertion may cause some structural change between the HAC centromere and the heterochromatin, both of which are responsible for the chromosome segregation and stability, and thought to be more dynamically changing through cellular senescence and between different types of cell lines (Maehara et al. 2010; Ohzeki et al. 2012). One possible explanation is that the differences of retention and transmission rates of the two HACs may be strongly influenced by epigenetic changes following mouse background conversion from BDF2 (ES) to B6 through the germ line. Although it is interesting to ask why an additional insertion decreases the stability of the circular HAC, we have already taken measures against it. Using the FLP-FRT system, HLA-DRA BAC and HLA-DRB1 BAC will be introduced into one expression cassette of the 25-4 HAC vector. We also developed two new site-specific recombination systems named VCre/VloxP and SCre/SloxP for genome engineering; the recognition sequences are different from the Cre recognition sequence (Suzuki and Nakayama 2011). These improvements enable us to introduce many BACs into one of chosen expression cassettes of the 25-4 HAC vector.

Although some instability was observed based on the manner of insertion, a de novo HAC vector carrying multiple genomic BACs was transmitted through at least eight generations, maintaining functional expression over seven generations, which is the minimum required for congenic breeding. We think that HAC vector technology opens up a new field that will offer advantages for generating transgenic animals that harbor multiple, large, single-copy DNA fragments and controlling the tissue-specific expression of transgenes.

## Electronic supplementary material

Below is the link to the electronic supplementary material.

Supplemental Fig. S1 Stability of the HLA genes on the HAC in mouse cells. Second filial generation: 5 PCR-positive F2 HLA-HAC pups were analyzed for two exons of HLA-DRA and two exons and two regions upstream of the start codon of HLA-DRB1. Lane 1 is positive control. Lane 2 is negative control. Lanes 3–7 are F2 samples. All sites analyzed in the 5 samples were positive, indicating that the inserted HLA-DRA and HLA-DRB1 genes were stably maintained through the HAC transfer into mouse. Eighth filial generation: The results of four samples are shown. Lane 1 is positive control. Lanes 2–5 are samples. Schematic diagrams show the positions of PCR targeted sites. This diagram was referenced on the NCBI gene browser (http://www.ncbi.nlm.nih.gov/gene). More than 120 mice through eight backcross generations were checked for the six positions of HLA-DRA and HLA-DRB1 on the HAC using genomic PCR. No deletion or different-sized bands were detected. These results show that the gene structures on the HAC in the mouse cells were stably maintained through the eight filial generations (GIF 38 kb)

High resolution image (TIFF 2956 kb)

Supplemental Fig. S2 Quantitative analysis of the expression of human transgenes in various tissues of TMC F8 mice (original data for Fig. 7) (GIF 52 kb)

High resolution image (TIFF 6887 kb)

Supplemental Fig. S3 RT-PCR analyses of mouse ES and CHO cells harboring HLA-HAC. Total RNA was isolated using the RNeasy Micro Kit (Qiagen). cDNA was synthesized using the Prime ScriptII 1st strand cDNA Synthesis Kit (TAKARA) and 25 ng aliquots used for PCR. The following primers were used: HLA-DRA gene, 5′-TCATAGCTGTGCTGATGAG-3′ and 5′- CAAAGCTGGCAAATCGTCC -3′; HLA-DRB1*0405 gene, 5′-AGCGGCGAGTCTATCCTGAG-3′ and 5′-AATGCTGCCTGGATAGAAAC-3′; beta-actin, 5′- GGCCCAGAGCAAGAGAGGTATCC -3′ and 5′- ACGCACGATTTCCCTCTCAGC -3′. The amplification conditions of HLA-DRA and HLA-DRB1 were 98 °C for 1 min, followed by 35 cycles of 98 °C for 10 s, 60 °C for 30 s, and 72 °C for 30 s. The PCR protocol for beta-actin was 94 °C for 4 min and 30 cycles of 94 °C for 30 s, 55 °C for 30 s, and 72 °C for 30 s (GIF 33 kb)

High resolution image (TIFF 4970 kb)

## References

[CR1] Altmann DM, Takács K, Trowsdale J, Elliott JI (1993). Mouse mammary tumor virus-mediated T-cell receptor negative selection in HLA-DRA transgenic mice. Hum Immunol.

[CR2] Asami J, Inoue YU, Terakawa YW, Egusa SF, Inoue T (2011). Bacterial artificial chromosomes as analytical basis for gene transcriptional machineries. Transgenic Res.

[CR3] Chandler KJ, Chandler RL, Broeckelmann EM, Hou Y, Southard-Smith EM, Mortlock DP (2007). Relevance of BAC transgene copy number in mice: transgene copy number variation across multiple transgenic lines and correlations with transgene integrity and expression. Mamm Genome.

[CR4] Doherty AM, Fisher EM (2003). Microcell-mediated chromosome transfer (MMCT): small cells with huge potential. Mamm Genome.

[CR5] Fukui Y, Esaki Y, Kimura A, Hirokawa K, Nishimura Y, Sasazuki T (1993). T-cell repertoire in a strain of transgenic C57BL/6 mice with the HLA-DRA gene on the X-chromosome. Immunogenetics.

[CR6] Gong S, Zheng C, Doughty ML, Losos K, Didkovsky N, Schambra UB, Nowak NJ, Joyner A, Leblanc G, Hatten ME, Heintz N (2003). A gene expression atlas of the central nervous system based on bacterial artificial chromosomes. Nature.

[CR7] Harrington JJ, Van Bokkelen G, Mays RW, Gustashaw K, Willard HF (1997). Formation of de novo centromeres and construction of first-generation human artificial microchromosomes. Nat Genet.

[CR8] Iida Y, Kim JH, Kazuki Y, Hoshiya H, Takiguchi M, Hayashi M, Erliandri I, Lee HS, Samoshkin A, Masumoto H, Earnshaw WC, Kouprina N, Larionov V, Oshimura M (2010). Human artificial chromosome with a conditional centromere for gene delivery and gene expression. DNA Res.

[CR9] Ikeno M, Masumoto H, Okazaki T (1994). Distribution of CENP-B boxes reflected in CREST centromere antigenic sites on long-range alpha-satellite DNA arrays of human chromosome 21. Hum Mol Genet.

[CR10] Ikeno M, Grimes B, Okazaki T, Nakano M, Saitoh K, Hoshino H, McGill NI, Cooke H, Masumoto H (1998). Construction of YAC-based mammalian artificial chromosomes. Nat Biotechnol.

[CR11] Ikeno M, Suzuki N, Hasegawa Y, Okazaki T (2009). Manipulating transgenes using a chromosome vector. Nucleic Acids Res.

[CR12] Ikeno M, Suzuki N, Kamiya M, Takahashi Y, Kudoh J, Okazaki T (2012). LINE1 family member is negative regulator of HLA-G expression. Nucleic Acids Res.

[CR13] Jux B, Staratschek-Jox A, Penninger JM, Schultze JL, Kolanus W (2013). Vav1 regulates MHCII expression in murine resting and activated B cells. Int Immunol.

[CR14] Kazuki Y, Oshimura M (2011). Human artificial chromosomes for gene delivery and the development of animal models. Mol Ther.

[CR15] Maehara K, Takahashi K, Saitoh S (2010). CENP-A reduction induces a p53-dependent cellular senescence response to protect cells from executing defective mitoses. Mol Cell Biol.

[CR16] Miyamoto K, Suzuki N, Sakai K, Asakawa S, Okazaki T, Kudoh J, Ikeno M, Shimizu N (2014). A novel mouse model for Down syndrome that harbor a single copy of human artificial chromosome (HAC) carrying a limited number of genes from human chromosome 21. Transgenic Res.

[CR17] Moro M, Cecconi V, Martinoli C, Dallegno E, Giabbai B, Degano M, Glaichenhaus N, Protti MP, Dellabona P, Casorati G (2005). Generation of functional HLA-DR*1101 tetramers receptive for loading with pathogen or tumour derived synthetic peptides. BMC Immunol.

[CR18] Nakayama M, Ohara O (2005). Improvement of recombination efficiency by mutation of Red proteins. Biotechniques.

[CR19] Ohtsuka M, Ogiwara S, Miura H, Mizutani A, Warita T, Sato M, Imai K, Hozumi K, Sato T, Tanaka M, Kimura M, Inoko H (2010). Pronuclear injection-based mouse targeted transgenesis for reproducible and highly efficient transgene expression. Nucleic Acids Res.

[CR20] Ohtsuka M, Miura H, Sato M, Kimura M, Inoko H, Gurumurthy CB (2012). PITT: pronuclear injection-based targeted transgenesis, a reliable transgene expression method in mice. Exp Anim.

[CR21] Ohzeki J, Bergmann JH, Kouprina N, Noskov VN, Nakano M, Kimura H, Earnshaw WC, Larionov V, Masumoto H (2012). Breaking the HAC barrier: histone H3K9 acetyl/methyl balance regulates CENP-A assembly. EMBO J.

[CR22] Pinkert CA, Widera G, Cowing C, Heber-Katz E, Palmiter RD, Flavell RA, Brinster RL (1985). Tissue-specific, inducible and functional expression of the E alpha d MHC class II gene in transgenic mice. EMBO J.

[CR23] Suzuki E, Nakayama M (2011). VCre/VloxP and SCre/SloxP: new site-specific recombination systems for genome engineering. Nucleic Acids Res.

[CR24] Suzuki N, Nishii K, Okazaki T, Ikeno M (2006). Human artificial chromosomes constructed using the bottom-up strategy are stably maintained in mitosis and efficiently transmissible to progeny mice. J Biol Chem.

[CR25] Suzuki M, Takahashi T, Katano I, Ito R, Ito M, Harigae H, Ishii N, Sugamura K (2012). Induction of human humoral immune responses in a novel HLA-DR-expressing transgenic NOD/Shi-scid/γcnull mouse. Int Immunol.

[CR26] Vintersten K, Testa G, Naumann R, Anastassiadis K, Stewart AF (2008). Bacterial artificial chromosome transgenesis through pronuclear injection of fertilized mouse oocytes. Methods Mol Biol.

