# Supplementary figures and images for: Generating a transgenic mouse line stably expressing human MHC surface antigen from a HAC carrying multiple genomic BACs
Source: Chromosoma. 2014 Oct 12;124(1):107–18. doi: 10.1007/s00412-014-0488-3 (PMC4339693; doi:10.1007/s00412-014-0488-3)

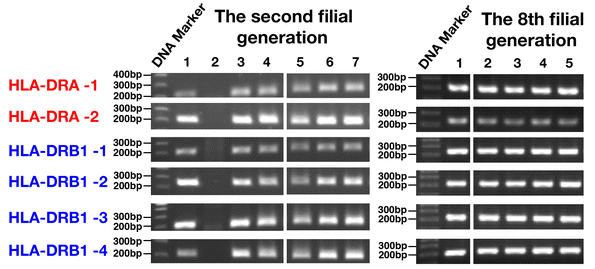

Supplement: Supplementary file 1 — Stability of the HLA genes on the HAC in mouse cells. Second filial generation: 5 PCR-positive F2 HLA-HAC pups were analyzed for two exons of HLA-DRA and two exons and two regions upstream of the start codon of HLA-DRB1. Lane 1 is positive control. Lane 2 is negative control. Lanes 3–7 are F2 samples. All sites analyzed in the 5 samples were positive, indicating that the inserted HLA-DRA and HLA-DRB1 genes were stably maintained through the HAC transfer into mouse. Eighth filial generation: The results of four samples are shown. Lane 1 is positive control. Lanes 2–5 are samples. Schematic diagrams show the positions of PCR targeted sites. This diagram was referenced on the NCBI gene browser (http://www.ncbi.nlm.nih.gov/gene). More than 120 mice through eight backcross generations were checked for the six positions of HLA-DRA and HLA-DRB1 on the HAC using genomic PCR. No deletion or different-sized bands were detected. These results show that the gene structures on the HAC in the mouse cells were stably maintained through the eight filial generations (GIF 38 kb) [file 412_2014_488_Fig8_ESM.gif]

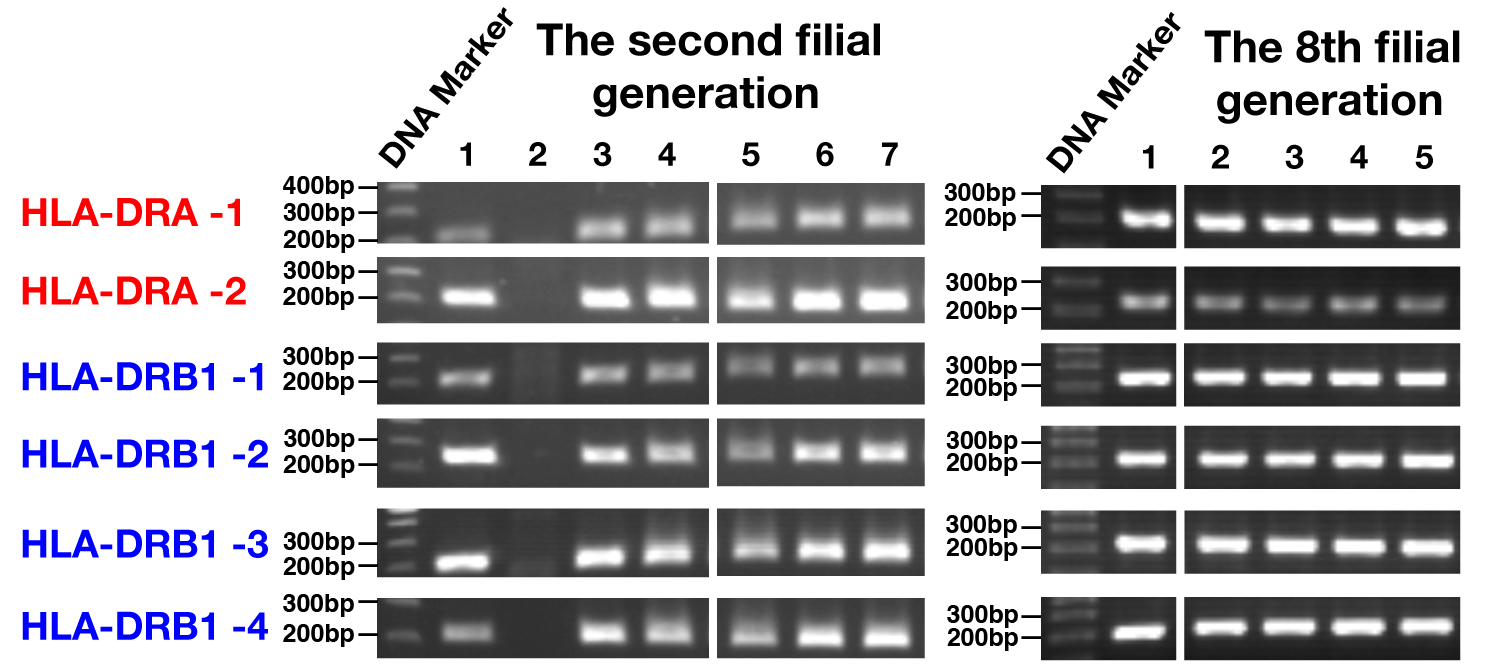

Supplement: Supplementary file 2 — High resolution image (TIFF 2956 kb) [file 412_2014_488_MOESM1_ESM.tif]

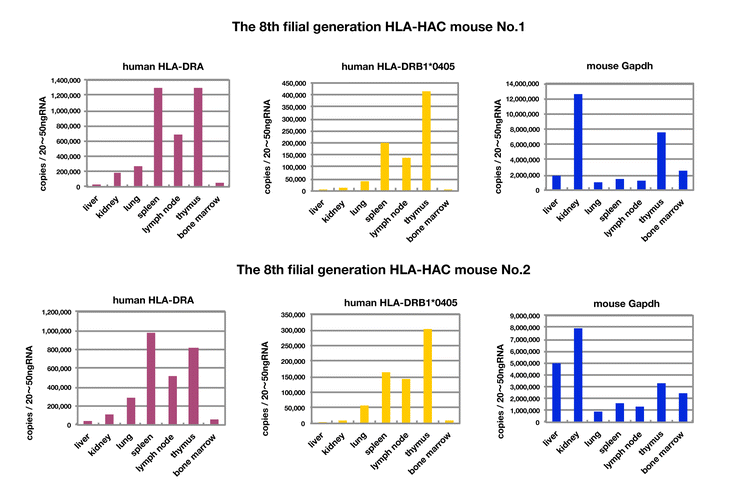

Supplement: Supplementary file 3 — Quantitative analysis of the expression of human transgenes in various tissues of TMC F8 mice (original data for Fig. 7) (GIF 52 kb) [file 412_2014_488_Fig9_ESM.gif]

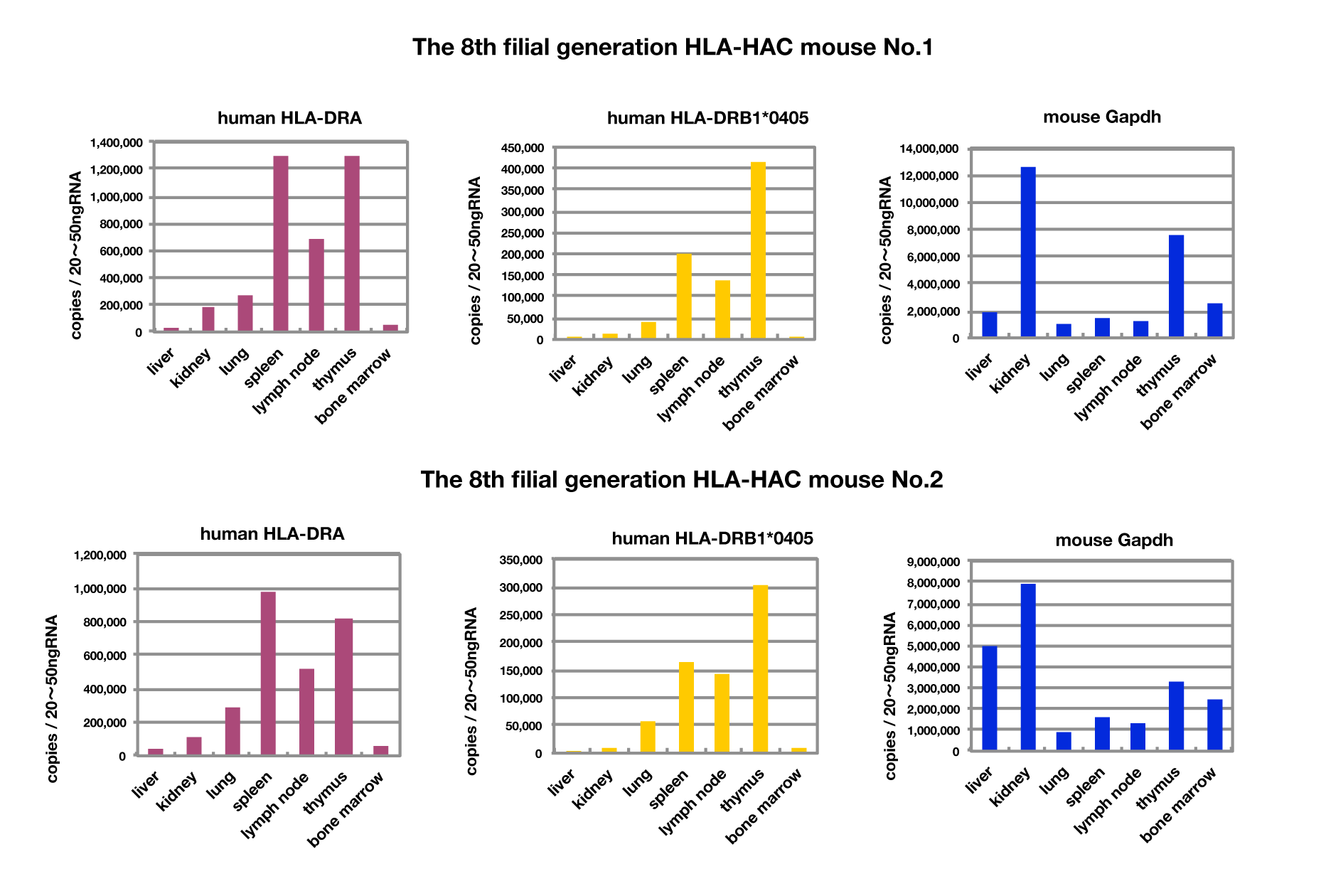

Supplement: Supplementary file 4 — High resolution image (TIFF 6887 kb) [file 412_2014_488_MOESM2_ESM.tif]

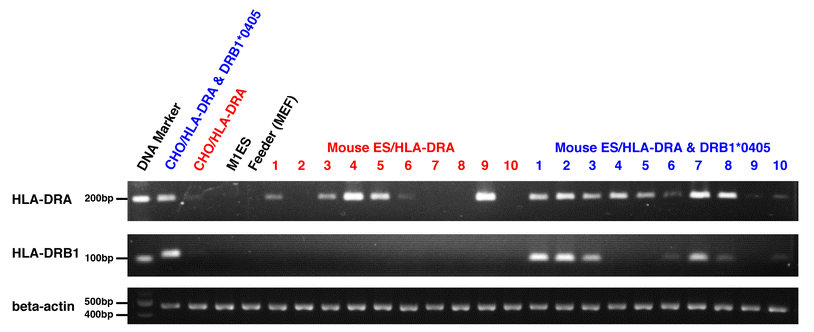

Supplement: Supplementary file 5 — RT-PCR analyses of mouse ES and CHO cells harboring HLA-HAC. Total RNA was isolated using the RNeasy Micro Kit (Qiagen). cDNA was synthesized using the Prime ScriptII 1st strand cDNA Synthesis Kit (TAKARA) and 25 ng aliquots used for PCR. The following primers were used: HLA-DRA gene, 5′-TCATAGCTGTGCTGATGAG-3′ and 5′- CAAAGCTGGCAAATCGTCC -3′; HLA-DRB1*0405 gene, 5′-AGCGGCGAGTCTATCCTGAG-3′ and 5′-AATGCTGCCTGGATAGAAAC-3′; beta-actin, 5′- GGCCCAGAGCAAGAGAGGTATCC -3′ and 5′- ACGCACGATTTCCCTCTCAGC -3′. The amplification conditions of HLA-DRA and HLA-DRB1 were 98 °C for 1 min, followed by 35 cycles of 98 °C for 10 s, 60 °C for 30 s, and 72 °C for 30 s. The PCR protocol for beta-actin was 94 °C for 4 min and 30 cycles of 94 °C for 30 s, 55 °C for 30 s, and 72 °C for 30 s (GIF 33 kb) [file 412_2014_488_Fig10_ESM.gif]

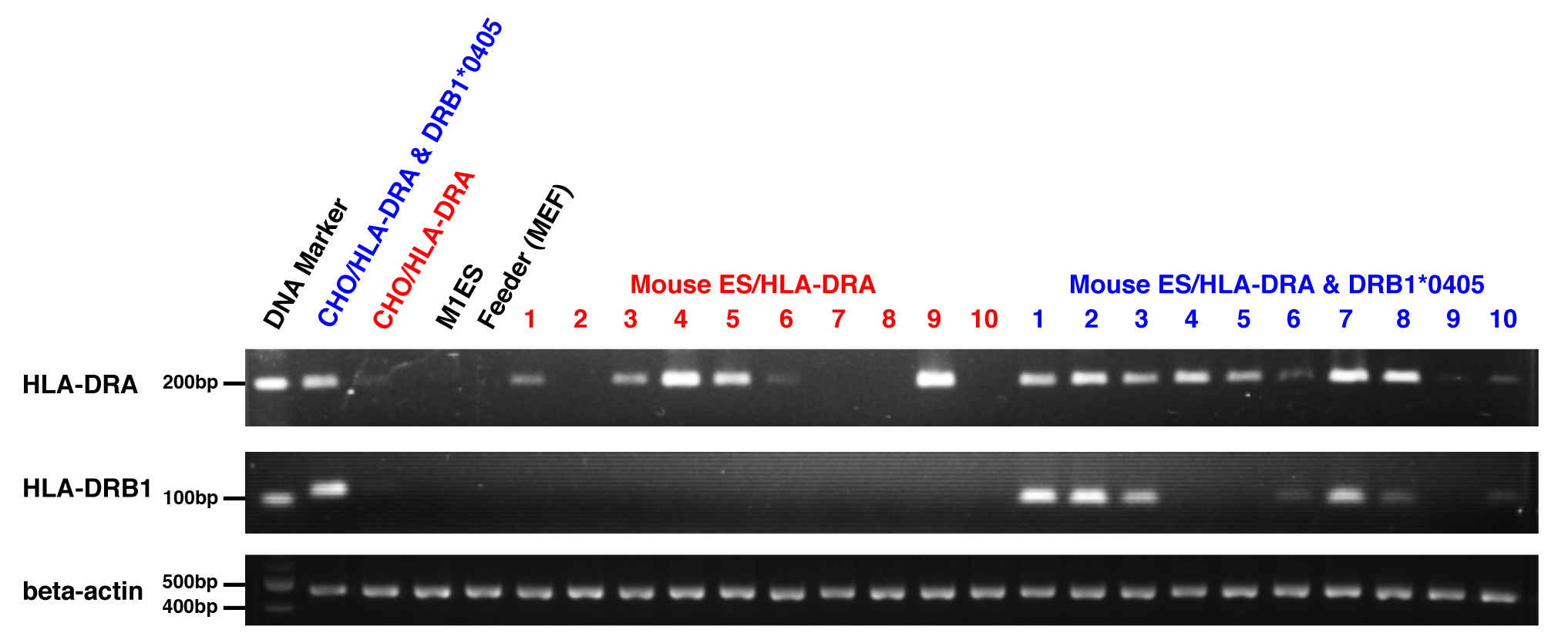

Supplement: Supplementary file 6 — High resolution image (TIFF 4970 kb) [file 412_2014_488_MOESM3_ESM.tif]
